# Occupational life-style programme over 12 months and changes of metabolic risk profile, vascular function, and physical fitness in blue-collar workers

**DOI:** 10.1186/s12995-023-00370-w

**Published:** 2023-03-22

**Authors:** Nina Schaller, Katharina Blume, Markus Hornig, Ludger Senker, Bernd Wolfarth, Tibor Schuster, Martin Halle, Katrin Esefeld, for the MOVING (MOtiVation, INtervention and Vascular chanGe) study group

**Affiliations:** 1 Department of Prevention, Rehabilitation and Sports Medicine, University Hospital (Klinikum rechts der Isar), Technical University of Munich, Georg-Brauchle-Ring 56, 80992 Munich, Germany; 2 Department of Sports Medicine, Humboldt-University, Charité University Medicine, Berlin, Germany; 3 Moving - Gesundheitsmanagement GmbH, Berlin, Germany; 4 Occupational Medicine Centers Northwest e.V. (Arbeitsmedizinische Zentren Nordwest e.V.), AMZ Lingen (Ems), Nordhorn, Germany; 5 BP Europa SE, Lingen Refinery, Lingen (Ems), Germany; 6 Department of Sports Medicine, Institute for Applied Scientific Training, Leipzig, Germany; 7 Department of Family Medicine, McGill University, Montreal, QC Canada; 8 DZHK (Deutsches Zentrum für Herz-Kreislauf-Forschung), Partner Site Munich, Munich Heart Alliance, Munich, Germany

**Keywords:** Occupational medicine, Cardio-metabolic risk, Prevention, Endothelium, Exercise

## Abstract

**Purpose:**

Occupational health programmes have been successfully implemented to improve body composition, physical fitness and cardiovascular risk. However, most programmes have been small and have not included long-term evaluation. Therefore, we evaluated a twelve-month life-style change programme in a German refinery.

**Methods:**

We offered a supervised six-week endurance exercise programme (2 × 90 min/week), starting after a two-day life-style seminar. After the active intervention and a half-day refresher seminar, employees were encouraged to continue exercising over one year on their own, with monthly supervised sessions to maintain adherence. Anthropometry, bicycle ergometry, cardio-metabolic risk profile, inflammatory parameters, and vascular function e.g. endothelial function was studied at baseline, after three and after twelve months.

**Results:**

Of 550 employees, *n* = 327 (age 40.8 ± 9.7 years, 88% males) participated in the study. Twelve-month intervention was associated with a reduced waist circumference (92.6 ± 12.2 to 90.8 ± 11.7 cm, 95% confidence interval for the mean change (CI): -2.5 to -1.1 cm) and a gain in maximal exercise capacity (202 ± 39.6 to 210 ± 38.9 Watt; 95% CI: + 5.1 to + 10.9 Watt). Metabolic and inflammatory parameters likewise HbA_1c_ and C-reactive protein improved in central tendency at a local 95% level of confidence. Vascular function e.g. Reactive-Hyperaemia-Index revealed a slight reduction, whereas no statistically robust changes in mean Cardio-Ankle-Vascular-Index and mean Ankle-Brachial-Index were observed.

**Conclusion:**

Health education added by a six-week supervised exercise programme was associated with minor long-term twelve-month improvements of body composition as well as physical fitness and a concomitant improvement of inflammatory state. These changes were, however, not clinically relevant and not accompanied by statistically robust improvements of vascular function.

**Trial registration:**

ClinTrialsGov: NCT01919632; date of registration: August 9, 2013; retrospectively registered.

## Introduction

Demographic changes of an increasingly older age of employees at workplace in combination with a deteriorating life-style behaviour including low physical activity and increasing prevalence of obesity and metabolic syndrome has raised serious concerns among companies regarding the maintenance of a healthy work force [1]. Therefore, increased attention has been given to effective occupational preventive health programmes [2–6]. The primary goal of these programmes is the improvement of overall health status of employees particularly across large companies, improvement of the social attitude and lowering costly rates of leave days, thereby maintaining established work flows and increasing productivity [7]. Moreover, the occupational setting is ideal in the sense that interventions can be offered by the company during or adjacent to working time [7]. Additionally, reimbursement may also be financially covered by the company itself or by health insurances, which have special overarching contracts with these companies.

On the one hand, this preventive concept of workplace health promotion (e.g. behaviour counselling, improving exercise/diet/non-smoking) has shown to be effective and data from larger studies have revealed beneficial effects on obesity/body weight, physical activity behaviour, physical fitness and cardiovascular risk factors [8, 9]. Likewise, Korshøj et al. (2016) revealed that lipid levels and inflammatory markers of blue-collar workers improved by a worksite aerobic exercise intervention (2 × 30 min /week) over 4 months. Reviews have shown strong evidence on body fat [10] and weight-related outcomes, especially for interventions with physical activity and/or nutrition [2], as well as in people with elevated risk for cardiovascular disease [10]. Furthermore, worksite interventions can also be effective in preventing mental and musculoskeletal disorders [2, 10, 11].

However, on the other hand, scientific evidence on long-term effects are limited or analyses have focused on standard body composition and cardiovascular parameters only [7]. Moreover, occupational intervention programmes addressing exercise only have been less applied in blue-collar workers, especially in those with a low socioeconomic status, which would benefit most from targeted interventions [7].

Therefore, we have conducted a one-year primary prevention exercise programme (“Moving”) for employees of an oil refining company mostly employing blue-collar workers. The objective was to examine the impact of an on-site programme on metabolic risk profile including inflammatory parameters as well as e.g. free fatty acid profile, exercise capacity assessed by maximal ergometry and on vascular function, i.e. endothelial function measured by the Reactive-Hyperaemia-Index (RHI) as primary endpoint. The hypothesis was that one year of participation in endurance exercise (twice a week for 90 min) may positively influence endothelial health.

## Methods

### Study design and population

The study has been designed as a prospective, one-arm, mono-centre and non-controlled intervention study. The group-based intervention consisted of an initial health-behaviour seminar followed by a supervised 6 weeks period and thereafter unsupervised exercise sessions over a total period of one year. During the whole programme, body weight, physical fitness as well as cardiovascular risk factors were monitored. The three medical appointments at baseline, at 3 and 12 months also included an assessment of endothelial function as a key parameter for vascular function [12].

The study was approved by the local ethics committee of the University Hospital “rechts der Isar”, Technical University of Munich, Germany (2555/09) in accordance with the Declaration of Helsinki. The study has been registered retrospectively and the trial registration number is NCT01919632. No changes have been made since commencement of the trial.

From 2009 to 2012, potential study participants were recruited by an oil refining company (BP Europa SE), from its site in Lingen (Ems), Germany, with a total of approximately 550 employees, mostly of them blue-collar workers. After announcement and advertisement by the company´s internal media, occupational medical department as well as CEO and executive board of BP Europa SE, Lingen Refinery, employees voluntarily signed up documenting their interest in participating in the study and the life-style programme. Inclusion criteria were: healthy men and women of all age, eligibility documented by the occupational medical department to participate in physical activity, written informed consent of study participation. Exclusion criteria included acute or chronic disease of any kind, which does not allow participation in physical activity, language or cognitive barriers that do not allow a communication about the study design and concept.

### Worksite health programme and life-style intervention

The initial stage of the worksite programme consists of a two-day health-behaviour seminar (^©^Moving - Gesundheitsmanagement GmbH, Berlin, Germany) in groups up to the size of 25 participants. In brief, on day one content included group-based life-style advice focusing on health prevention strategies, role of physical (in-)activity and cardiovascular risk. Day two covered topics like healthy nutrition, behaviour change management, self-motivation strategies, and resistance exercise training.

After this prelude seminar, the groups started into a supervised outdoor exercise programme on the site of the refinery using the area of and around the factory grounds. Over a period of six weeks, experienced and instructed exercise instructors instructed and supervised all training sessions (twice a week for 90 min) of endurance exercise (i.e. jogging, and Nordic walking) in groups. Concepts included fundamental exercise behavioural strategies like 1.) “start-low, go slow”, 2.) First increase of duration, thereafter intensity, 3.) Combination of endurance, resistance and flexibility and coordination exercises. After this time, a half-day refresher seminar was held and participants received recommendations to continue this exercise programme identically but unsupervised for up to one year. The employees were, however, encouraged to continue meeting in the same groups. Monthly supervised exercise training sessions with the same instructors (10 sessions over 46 weeks) were added to the unsupervised intervention phase in order to improve motivation and maintain adherence even beyond the supervised phase. General nutritional advice was given at the initial and refresher seminar, but not in an individual setting.

**Fig. 1:**
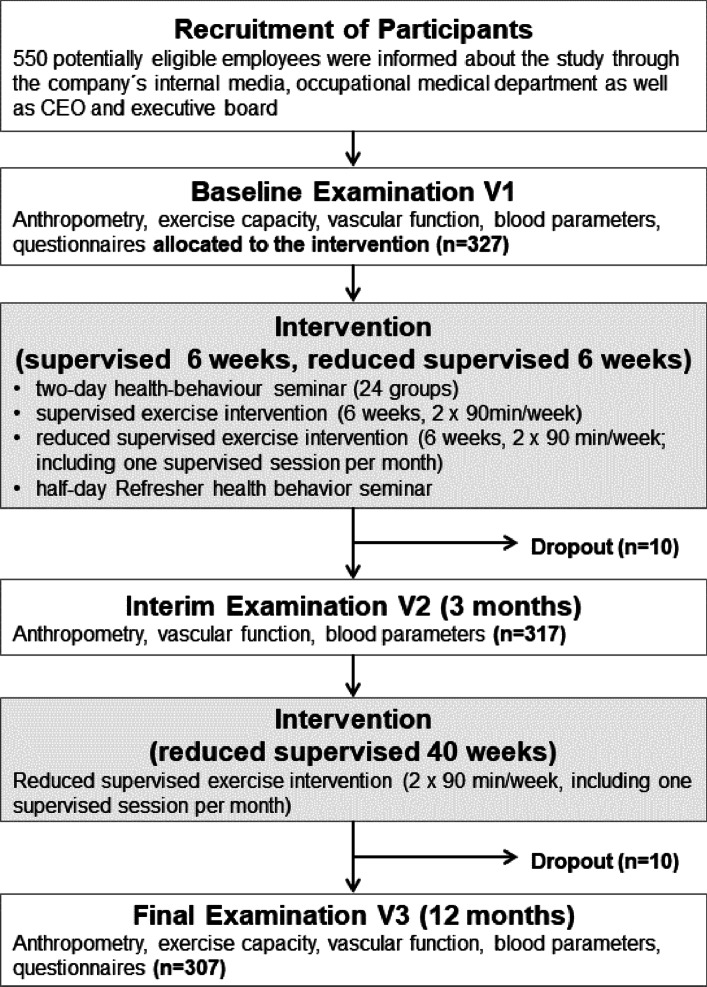
Flow diagram of recruitment, examinations and intervention

Clinical investigations were performed locally by the occupational medical department of the refinery after being instructed by external staff of the department of prevention and sports medicine of the Technical University of Munich, Germany. These examinations were performed at baseline (V1) before the seminar, after three months (V2) and after 12 months (V3) (Fig. 1). As a primary parameter, endothelial function was measured by finger plethysmography (^©^EndoPAT, itamar, Italy) after induction by upper-arm occlusion and changes were expressed as Reactive-Hyperaemia-Index (RHI) [13]. Secondary parameters included vascular function parameters CAVI (Cardio-Ankle-Vascular-Index), ABI (Ankle-Brachial-Index) and AI (Augmentation Index). Moreover, maximal exercise capacity (Watt/kg) was assessed by bicycle ergometry at V1 and V3. Anthropometric parameters (body weight, body-fat and waist-circumference) and systolic as well as diastolic blood pressure was assessed at all three time-points. Clinical chemistry at all three time-points included inflammatory and metabolic parameters, e.g. blood glucose, HbA_1c_, total cholesterol, HDL, LDL, triglycerides, C-reactive protein and Omega-3-Index [14]. Also, lifestyle and health status were assessed by questionnaire at V1, V2, and V3. The PROCAM (Prospective Cardiovascular Munster) score [15] was calculated to predict and evaluate the 10-year cardiovascular risk. Data from the baseline and after twelve months are reported in this manuscript.

### Statistics

This is a prospective, mono-centre, non-controlled intervention study. Data was analysed in an exploratory manner. Descriptive statistics were performed reporting mean values, median, standard deviation, minimal and maximal values as well as number of patients per analysis. Since sample size was sufficiently large (*n* > 30) to obtain asymptotically robust results, Student’s t-test for paired samples was applied to assess mean changes in outcome measures between baseline and 12 months follow up. No correction of type I error probability was applied and hence, unadjusted p-values and 95% confidence intervals were reported as exploratory distance and precision indices. For statistical analysis the programme SPSS Version 26.0 for Windows was used.

## Results

### Participants

Between 2009 and 2012, 327 employees (age 40.8 ± 9.7 years, 88% males) were assigned to 14 separate groups. Assuming a total workforce of approximately 550 employees, this corresponds to a participation rate of 60%. Because of the gender structure of an oil refinery, more males than females were recruited (87.5% vs 12.5%). About half of the participants reported to work partly in shift work (48.2%), night work (48.2%) and on weekends (55.8%). 71.3% of the participants worked mainly sedentary, whereas 27.7% worked mainly physically demanding. The baseline characteristics of the participants are listed in Table 1.

**Table 1:** Baseline characteristics of the participants

Characteristics	Participants
Male/Female (*n* = 327) (%)	286/41 (87.5/12.5)
Age, years (*n* = 327)	40.8 ± 9.7 (42.0 / 20–60)
Smoking (*n* = 327) (%)	27 (8.3)
Mainly physically inactive (*n* = 301) (%)	39 (13.0)
Medication, Hypertension (*n* = 303) (%)	28 (9.2)
Medication, Hypercholesterolaemia (*n* = 303) (%)	15 (5.0)
Shift work (*n* = 301) (%)	145 (48.2)
Night work (*n* = 301) (%)	145 (48.2)
Weekend work (*n* = 301) (%)	168 (55.8)
Mainly computer work (*n* = 303) (%)	216 (71.3)
Mainly physical demanding work (*n* = 303) (%)	84 (27.7)

Data for categorical variables are presented as numbers (n) and percentages (%)

Data for continuous variables are presented as mean ± standard deviation (median / minimum – maximum)

Data from 317 participants could be followed at three months and 307 participants at 12 months (Fig. 1). Regarding adherence of the supervised training sessions, the trainers reported a training adherence of 68% of the first six weeks supervised phase and 42% in the following weeks with reduced supervision.

### Anthropometry

Weight, body mass index (BMI) and body fat remained stable after 12 months. Waist circumference decreased (-1.8 cm, 95% CI: -2.5 to -1.1) (Table 2).

**Table 2:** Comparison of anthropometry, cardiovascular risk factors, physical fitness and vascular function between baseline examination and final examination after 12 months

	**n**	**Baseline**			**After 12 months**			***p*-value**	**95% CI** **mean change**
**Anthropometry**									
Weight (kg)	291	88.5	±	15.6	88.1	±	15.0	0.060	-0.9; + 0.02
Waist Circumference (cm)	291	92.6	±	12.2	90.8	±	11.7	< 0.001	-2.5; -1.1
Body Mass Index (kg/m^2^)	291	27.0	±	4.0	26.9	±	3.8	0.085	-0.3; + 0.02
Body Fat (%)	291	21.3	±	4.9	21.5	±	4.9	0.232	-0.2; + 0.6
**Risk factors**									
Haemoglobin A_1c_ (%)	292	5.6	±	0.4	5.5	±	0.4	< 0.001	-0.2; -0.09
Glucose (mg/dl)	291	78.3	±	13.2	81.6	±	15.5	0.001	+ 1.4; + 5.2
C-reactive Protein (mg/dl)	292	0.39	±	0.38	0.16	±	0.28	< 0.001	-0.28; -0.18
Uric Acid (mg/dl)	294	5.8	±	1.2	5.8	±	1.1	0.428	-0.1; + 0.2
Ferritin (mg/dl)	294	137.3	±	128.9	128.4	±	119.6	0.004	15.1; -2.9
Triglycerides (mg/dl)	294	122.7	±	67.6	120.0	±	84.6	0.485	-10.4; + 4.9
Total Cholesterol (mg/dl)	294	205.1	±	38.4	204.6	±	38.5	0.754	-3.6; + 2.6
Low Density Lipoprotein (mg/dl)	294	131.1	±	36.5	130.0	±	35.6	0.421	-4.1; + 1.7
High Density Lipoprotein (mg/dl)	294	53.7	±	13.5	54.3	±	13.4	0.242	-0.4; + 1.6
Omega-3-Index (%)	219	5.5	±	1.3	5.5	±	1.4	0.335	-0.2; + 0.06
10-year cardiovascular risk (%) by PROCAM score)	286	3.7	±	4.9	3.1	±	3.6	0.006	-1.0; -0.2 -
**Physical fitness**									
Heart Rate, at rest (bpm)	290	67.1	±	11.6	66.8	±	12.9	0.652	-1.7; + 1.1
Heart Rate, maximal (bpm)	287	165	±	17.4	167	±	16.6	0.060	-0.07; + 3.3
Maximal exercise capacity (Watt)	287	201.7	±	39.6	209.8	±	38.9	< 0.001	+ 5.1; + 10.9
Relative exercise capacity (Watt/kg)	287	2.3	±	0.50	2.4	±	0.5	< 0.001	+ 0.1; + 0.13
**Vascular function**									
Reactive-Hyperaemia- Index	286	2.00	±	0.72	1.87	±	0.49	0.007	-0.2; -0.04
Cardio-Ankle-Vascular-Index	287	6.98	±	1.32	7.07	±	1.48	0.341	-0.96; + 2.8
Ankle-Brachial-Index	290	1.17	±	0.11	1.17	±	0.11	0.892	-1.6; + 1.4
Systolic blood pressure (mmHg)	290	140.6	±	16.0	140.7	±	14.6	0.966	-1.6; + 1.6
Diastolic blood pressure (mmHg)	290	87.6	±	10.0	88.4	±	9.9	0.115	-0.2; + 1.9

Data are shown as mean ± standard deviation

*CI* Confidence interval

*p*-values and 95% confidence intervals are based on two-sided Student t-test for paired samples (no correction for Type I error inflation due to multiple testing applied); *PROCAM* Prospective Cardiovascular Munster

### Risk factors

The metabolic and inflammatory parameters HbA_1c_ and C-reactive protein improved over the 12 months follow-up period, whereas changes in uric acid, blood lipids and Omega-3-Index did not change. Fasting glucose (*p* = 0.001) and ferritin (*p* = 0.004) slightly deteriorated. The 10-year cardiovascular risk by PROCAM improved by 0.6% (95% CI: -1.0 to -0.2%) (Table 2).

### Physical fitness

Bicycle ergometry revealed that maximal exercise capacity improved by + 8.1 W (95% CI: + 5.1 to + 10.9 W). Resting and maximal heart rate remained largely unchanged (Table 2).

### Vascular function

Endothelial function analysed as Reactive-Hyperaemia-Index revealed a slight decrease (-0.13, 95% CI: -0.0.2; -0.04), whereas no systematic changes in blood pressure, Cardio-Ankle-Vascular-Index and Ankle-Brachial-Index could be established with sufficient statistical confidence (Table 2).

## Discussion

Introducing a short-term life-style programme with exercise intervention as the core approach within a worksite prevention strategy initiative was associated with long-term minor improvements of physical fitness, abdominal obesity, systemic inflammation, glucose metabolism as well as 10-year-cardiovascular risk by PROCAM (Table 2). Of note, this intervention only consisted of a two-day seminar focusing on behavioural changes, a six weeks supervised exercise intervention twice weekly, a follow-up seminar of a half day and encouragement to continue exercise intervention, which was supported by monthly supervised exercise sessions. Data shown are the result of a long-term assessment after one year, far beyond the active intervention phase of six weeks. Moreover, the programme was introduced in a large proportion of employees involving 60% of the whole work force at one company, which is, as a refinery, not per se a classical preventive strategies open employee cohort, characterized by a proportion of 50% working night or weekend shifts (Table 1). However, the achieved changes were not clinically relevant and no systematic changes of vascular function parameters, lipids or fatty acid profile could be established in this healthy sample (Table 2).

With regard to the relatively small effects on metabolic and vascular components after 12 months, it has to be taken into account that the study participants were a relatively healthy cohort (10-year cardiovascular risk at baseline: 3.7%; smoking rate at baseline: 8.3%). These findings are in line with previous studies and systematic reviews [7, 16]. Workplace interventions have shown to reduce body weight and waist circumference, but associations with biochemical markers as well as on blood pressure were inconclusive and small, respectively [17]. An ecological study, which examined trends of cardiovascular risk factors from 2008 – 2017 in oil refinery workers, found increasing rates only of hypertension and diabetes. Rates of low HDL, high LDL, high cholesterol, smoking and coronary artery risk decreased. The authors stated a medium cardiovascular risk in oil refinery workers and called for systematic health promotion at the workplace [18].

However, when starting with abnormal baseline levels, improvements might be far larger. In our study, with regard to endothelial function measured by RHI as primary endpoint, mean baseline values of RHI 2.00 ± 0.72 and RHI 1.87 ± 0.49 after 12 months were low, but still were above the cut-off value for endothelial dysfunction of RHI ≤ 1.67 [13, 19]. Previous studies revealed little effect of exercise training on endothelial function in healthy individuals, but endothelial function improved particularly in those with abnormal baseline endothelial function [20].

Nutritional advice or counselling beyond the introductory seminar was not given, which may explain that body weight was not considerably reduced over 12 months. Moreover, no changes of the Omega-3-Index, a parameter dependent on the consumption of unsaturated fatty acids, was observed (Table 2), although values were very low at baseline [14]. Therefore, individual nutritional counselling could have been a valid prevention strategy in addition to exercise intervention, as has shown by previous investigations [21–23]. We, however, did not include individual nutritional advice into our programme, as it was reported from previous experience by the corporate medical department and the employee representation that the employees were reluctant to adopt changes in nutrition to a large extend. It was felt that focussing on exercise intervention was more feasible and long-lasting. This approach was confirmed by our 12-months programme results that exercise training was only associated with improved exercise capacity also long-term. These findings are beyond most studies and even our experience that improvements observed during supervised intervention can mostly not be maintained during follow-up [24]. Obviously, the monthly training sessions offered onsite the factory area seem to be a practical and successful approach for maintaining adherence to exercise programmes. In a sub-analysis (data not shown) we classified the trainings adherence of all participants to low, moderate and high. In all three groups there was a slight reduction of 0.5% to 0.7% in the 10-year cardiovascular risk by PROCAM after 12 months.

In general, physical activity have multiple health benefits, particularly on metabolic control, vascular function and cardiovascular morbidity and mortality and is strongly recommended in recent guidelines [25, 26]. However, there seems to be an exercise paradox when comparing exercise performed during leisure time or during the occupational setting. Data from the Copenhagen General Population Study with 104,046 adults revealed that higher leisure time physical activity is associated with a 15% reduction of risk for major cardiovascular events (MACE) and a 40% reduction of all-cause mortality risk [27]. At the same time, higher occupational physical activity was associated with a 35% increase of MACE risk and a 27% increase of risk for death [27]. These paradoxical results may be explained by the different characters of leisure and occupational physical activities and by occupational various stressors, e. g. shift work [28].

Regarding monetary benefits, occupational health programmes offer a lot of potential. However, cost effectiveness of corporate wellness programmes has been challenged in a recent review [16]. The economic impact of the programmes is hard to monetise. Given the small changes and unknown cost implications, the economic evidence of workplace-interventions remains uncertain. We did not apply any systematic cost-analysis, but as we have calculated approximately €300 for each individual for one year including seminars and exercise sessions, we are convinced that this programme is cost-effective. This amount, however, does not include risk assessment examinations.

### Limitations

The strongest limitation is the lack of a control group. Consequently, no causal conclusions may be drawn from the results. However, the changes during intervention are certainly induced by the life-style changes, while a control group would perhaps even deteriorate. Nonetheless, a randomized trial would have been optimal. Regarding inclusion, maybe there is a selection bias, because health-conscious people are more likely to apply to health promotion programmes. Moreover, inclusion of more females would have been relevant to see changes in that group and compare these with male counterparts. However, females are not represented at an oil refinery because of the heavy labour of most jobs. Therefore, the number was overall low. Nonetheless, the strength of the current study is that 60% of employees participated in the 12 months programme and adherence was high.

In our experience this is mainly linked to the strong support of the company´s executive board in combination with the internal media and occupational health department. The health seminar added by medical examinations was clearly seen as an incentive for workers, which improved adherence to the programme. Previous studies have shown that unionization and management support were the strongest predictors of the adoption of health programmes [29].

## Conclusion

The results of the evaluation and personal experience performing the Moving programme imply that it is a feasible and successful practice example. The introduction of a behavioural seminar including education and advice over two days regarding medical background and practical applications of life-style measures followed by supervised exercise for six weeks was associated with minor long-term twelve-month improvements in body composition, physical fitness and inflammatory state. In this healthy sample, these changes were, however, not clinically relevant and not accompanied by statistically robust improvements of vascular function. Targeting especially those employees high at risk with a worksite exercise intervention would be more effective, and thus could be highly cost-effective for companies and health insurances in the long-term [7, 28].

## Data Availability

The datasets generated during and/or analyzed during the current study are not publicly available due to data protection regulations of our clinic, but are available from the corresponding author on reasonable request, only in accordance with the data protection regulations of our clinic.
